# Levels of heavy metals in the raw and processed Ethiopian tobacco leaves

**DOI:** 10.1186/s40064-016-1770-z

**Published:** 2016-02-29

**Authors:** Girma Regassa, Bhagwan Singh Chandravanshi

**Affiliations:** Department of Chemistry, College of Natural Sciences, Addis Ababa University, P.O. Box 1176, Addis Ababa, Ethiopia; Department of Chemistry, Madawalabu University, P.O. Box 247, Robe, Bale, Ethiopia

**Keywords:** Tobacco leaves, Cigarette tobacco leaves, Heavy metals, Nyala, Ethiopia

## Abstract

Tobacco (*Nicotiana tabacum* L.) is a commercial plant. Tobacco leaves naturally accumulate and concentrate relatively high levels of heavy metals and particular cadmium in leaves. Tobacco is one of the basic agricultural products, in Ethiopia, with social and economic importance. However, there is no report in the literature on the determination of levels of heavy metals in Ethiopian tobacco leaves. Hence this research is intended to determine the levels of heavy metals in the raw and processed Ethiopian Virginia tobacco leaves. Samples of raw Virginia tobacco leaves were collected from two different regions of Ethiopia (Billate and Shewa Robit). The three processed tobacco samples were collected from National Tobacco Enterprise, Addis Ababa, Ethiopia. The samples were wet-digested using 3 mL HNO_3_ (69–72 %) and 3 mL HClO_4_ (70 %) at 350 °C for 3.5 h and concentrations of heavy metals (Cd, Cr, Cu, Ni, Pb and Zn) in the samples were determined by flame atomic absorption spectrometer. The mean metals concentrations (in µg/g dry weight) in the raw Virginia tobacco leaves from Billate and Shewa Robit, respectively, were: Cu (4.38, 7.30), Zn (53.7, 33.2), Cd (1.20, 1.30), Cr (ND, 1.45), Ni (ND, 1.90). The mean metals concentrations (in µg/g dry weight) in the processed tobacco from Billate and Shewa Robit, respectively, were: Cu (9.80, 12.8), Ni (2.35, 2.20) Cd (1.45, 1.90), Cr (1.65, 1.75), Zn (101, 83.8). The mean metals concentrations (in µg/g dry weight) in the processed tobacco Nyala (Ethiopian cigarette leaves) were: Cu (8.95), Cd (1.55), Cr (1.62), Ni (4.70), Zn (79.3). The concentrations of Cr and Ni in tobacco leaves from Billate and Pb in all the tobacco samples were below the detection limits. This study showed that the metal contents of tobacco leaves varied with the geographical origin in which the tobacco plant grows. The metal contents of processed tobacco were higher than the corresponding raw leaves. Pb was not detected in both the raw and processed Ethiopian tobacco leaves.

## Background

Tobacco (*Nicotiana tabacum* L.) is a commercial plant. Tobacco is one of the basic agricultural products, in Ethiopia, with social and economic importance. Native tobacco (‘gaya’) has been used for snuffing, chewing and for pipe smoking in many places.

Tobacco is grown for commercial purposes by state-owned farms and by farmers around these farms. The National Tobacco Enterprise has been given the mandate to organize tobacco production and processing in the country. Three main types of commercial tobacco are produced in Ethiopia: Virginia, Oriental and Burley. Virginia accounts for a little more than 74 % of the total production, followed by Oriental, 22 %, and Burley, 4 % (FAO 1995, 2003). Major cultivation areas are Sidamo, Northern Shoa, and Hararghe. Commercial production is concentrated in Shewa Robit (North Shoa), Billate, Awassa, Wolaita (Sidamo) and in Nura-Era (Hararghe). State farms in these areas produce about 500–900 MT of cured leaves annually from about 2000 hectares of land (FAO 1995, 2003).

UNODC report (Gebre Selassie and Gebre 1996) reveals that in Ethiopia peoples commonly use khat, tobacco, and alcohol, which have a share of 48.2, 29.9 and 18.9 %, respectively, of all type of drugs. This clearly shows that tobacco made significant contribution as a drug in Ethiopia. Commonly Virginia, Oriental and Burley are commercial tobacco species used for making cigarettes in Ethiopia.

Tobacco (*Nicotiana tabacum* L.) is a commercial plant. Tobacco leaves are used for cigarette production and chewing. It naturally accumulates and concentrates relatively high levels of heavy metals and particular cadmium in leaves (Kaličanin and Velimirović 2012; Ajab et al. 2014). Cadmium is a non-essential to both plant and human. It is highly toxic and gets accumulated by tobacco plants. Cadmium is transferred to human being through cigarette smoking (Jarup et al. 1998; Nordberg et al. 2007; Verma et al. 2010). Most heavy metals cause a significantly serious damage on human health (Baldwin and Marshall 1999; Smith and Doolittle 1999; Health Canada 1999; Rothwell and Yorkshire 1999; Stojanovic et al. 2004; Sharma and Dubey 2005; Nnorom et al. 2005; Zhang et al. 2005; Lugon-Moulin et al. 2006). Cadmium is associated with bone and kidney diseases and Pb with neurological disorders (Sharma and Dubey 2005; Nnorom et al. 2005). Excess of Cu and Zn are associated with metabolic disorders potentially resulting in death (Stojanovic et al. 2004; Zhang et al. 2005). One of the main sources of toxic metals in our environment is tobacco smoke. Cigarette smoking is a major source of intake of these toxic elements not only to the smoker but also, through passive smoking, to nonsmokers.

The degree of accumulation of heavy metals in the tobacco plant results from a complex interaction between the soil and plant. The degree of accumulation of the metals from the soil depends on the type of soil, the pH value, the quality of water used for irrigation, the chemical composition of the metal, and the type of tobacco plant (Grant et al. 1998; Golia et al. 2008). The farmers use large amounts of fertilizers and pesticides during the production of tobacco plant. The fertilizers and pesticides usually contain high concentrations of metals and contribute a major degree in the pollution of agricultural soil, as well as plants (Karaivazoglou et al. 2007; Lecours et al. 2012).

The distribution and accumulation of metals in tobacco leaves are the reflection of the mineral composition of the soil and environment in which the tobacco plant grows. Therefore, the actual metals content of tobacco vary considerably according geographic origin, the use of fertilizers with different chemical compositions and other characterizing features such as water for irrigation (Peuarossa et al. 1990; Adeyeye 2005; Lugon-Moulin et al. 2006). The phosphates fertilizers, which are used in tobacco cultivation, contain high concentrations of heavy metals.

Literature survey revealed a large number of report on the determination of two of the most toxic heavy metals (As and Hg). Arsenic concentration averaged (0.4 ± 0.6 μg/g) in the tobacco samples from Africa, Asia, Europe, South and North America has been determined by inductively coupled plasma-mass spectrometry (Lugon-Moulin et al. 2008). Taebunpakul et al. (2011) have determined total arsenic and arsenic speciation in tobacco products: from tobacco leaf and cigarette smoke by HPLC-ICP-MS. Lazarević et al. (2012) have determined the contents of arsenic in tobacco (<0.02–2.04 μg/g) and cigarettes (<0.02–0.71 μg/g) by electro-thermal atomic absorption spectrometry. Campbell et al. (2014) have determined total inorganic arsenic species (144–3914 μg/kg) in tobacco samples from different origin using HPLC-ICP-MS. Piadé et al. (2015) have determined arsenic levels in tobacco filler and cigarette smoke in a large number of samples worldwide survey and reported median tobacco level for arsenic 237 ng/g while median mainstream smoke yields for arsenic was <3.75, ng/cigarette. Maruyama and Komiya (1973) have determined arsenic and mercury in tobacco leaves by neutron activation analysis. Suzuki et al. (1976) have determined mercury in cigarettes. Chang et al. (2002) reported a rapid method for the determination of mercury in mainstream cigarette smoke by two-stage amalgamation cold vapor atomic absorption spectrometry and the vapor phase mercury in the mainstream smoke was found 7.4 ± 0.4 ng/cigarette. Zhangyu et al. (2004) have reported levels of mercury in tobacco and tobacco additives by microwave digestion and RP-HPLC followed by on-line column enrichment. Kowalski and Wierciński (2009) have reported mercury contents in cigarette tobacco (6.48–10.56 ng/g, 2.95–10.2 ng Hg per a single cigarette) determined using mercury analyser.

Several studies have also been reported in the literature on determinations of the levels of heavy metals in the raw and processed tobacco leaves (Kaličanin and Velimirović 2012; Eneji et al. 2013; Ajab et al. 2014) in the different parts of the world using different techniques (Saldivar et al. 1991; Health Canada 1999; Stojanovic et al. 2004; Angelova et al. 2004; Zhang et al. 2005; Nnorom et al. 2005). Some studies have also been carried out in Ethiopia on the levels of essential and non-essential metals in a psychoactive khat leaves (Atlabachew et al. 2010, 2011) as well as in cannabis leaves (Zerihun et al. 2015). A few studies have also been carried out on the levels of nicotine in Ethiopian tobacco leaves (Geto et al. 2012; Kassa et al. 2013; Tassew and Chandravanshi 2015). However, to the best of our knowledge, there is no report in the literature on the levels of heavy metals in Ethiopian tobacco leaves. Hence this research is intended to determine levels of six heavy metals, four of them are toxic (Cd, Ni, Pb, Cr) and two of them are essential (Cu, Zn), in the raw and processed Ethiopian tobacco leaves by flame atomic absorption spectrometry. Two of the most toxic metals (As and Hg) were not determined in this study because their determination require highly sensitive and more sophisticated methods and instruments such as neutron activation analysis, cold vapor atomic absorption spectrometry, hydride generation atomic absorption spectrometry, electrothermal atomic absorption spectrometry and inductive couple plasma-mass spectrometry.

## Methods

### Instrument and apparatus

Ceramic pestle and mortar were used for grinding and homogenizing of the raw and processed tobacco leaves. Digital analytical balance (Mettler Toledo, Model AT250, Switzerland) and oven (Digitheat, J.P. Selecta, Spain) were used for weighing and drying the samples, respectively. Quick-fit round bottom flasks (150 mL) fitted with reflux condenser were used in Kjeldahl apparatus hot plate to digest the samples. Buck Scientific Model 210VGP (East Norwalk, USA) atomic absorption spectrophotometer equipped with deuterium ark background correctors and air–C_2_H_2_ flame was used for the determination of heavy metals.

### Chemicals, reagents and standard solutions

Chemicals and reagents used for the analysis were of analytical reagent grade. 69–72 % HNO_3_ and 70 % HClO_4_ (Analar^®^, BDH, England) were used for digestion of tobacco samples. Stock standard solutions of the metals (Zn, Cr, Cu, Ni, Pb, and Cd), 1000 mg/L calibration standards (Buck Scientific, USA), prepared as nitrates for each element in 2 % HNO_3_, were used for the preparation of calibration curves for the determination of metals in the samples. Distilled-deionized water was used for preparation of standard solutions and dilution.

### Description of sample sites

Samples were collected from two-tobacco plantation areas (Shewa Robit and Billate), which account for more than 76 % of total tobacco plant production in Ethiopia and are the only places where Virginia type tobacco is planted. Billate is located in Southern Ethiopia 300 km from Addis Ababa. Shewa Robit is located in the Northern Ethiopia 215 km from Addis Ababa. Both places are located in East African Rift Valley. Therefore, the two areas have more or less similar weather conditions and climate. National Tobacco Enterprise, which is the only factory for manufacturing cigarette in Ethiopia, operates a cigarette factory located in Addis Ababa. For this study, Virginia type tobacco was chosen because of its high availability and Nyala (Ethiopian cigarette) type cigarette which is totally manufactured from Virginia type tobacco and accounts more than 89 % of total cigarette production in Ethiopia (Food and Agricultural Organization 1995; National Tobacco Enterprise 2006).

### Sample collection

Depending on the availability of tobacco plant, representative amount of leaves, four leaves per plant staring from bottom to the tips by stalk position, from Billate and Shewa Robit were collected. The collected samples were washed with tap water and rinsed three times with deionized water to make them free of extraneous substances, including soil and dust particles, and foliar spray residues that may influence analytical results (Jones et al. 1991; Maier and Griepink 1995; Iyengar et al. 1998). Another three samples were, processed Virginia whose origin was Billate, Shewa Robit, and Nyala sample. These processed samples, which were ready for use after warping, were prepared in the laboratory of the cigarette factory in exactly the same procedure of Nyala manufacturing by an expert of laboratory in the factory. Both types of samples were sealed in washed, rinsed with distilled-deionized water and dried polyethylene bag and transported to the laboratory where further sample pre-treatments were made. Drying at temperatures under 80 °C may not remove all combined water and may result in poor homogenization and incorrect analytical results. Drying temperatures above 80 °C may result in thermal decomposition and reduction in dry weight (Jones et al. 1991). Accordingly, after chopping the samples with plastic knife, all the samples were dried in the oven for 24 h at 80 °C. The dried samples were well ground by using pestle and mortar and kept in the desiccator until digestion (Nnorom et al. 2005).

### Digestion of tobacco samples

Applying the optimized procedure, 0.5 g of well-powdered tobacco sample was digested with 3.0 mL HNO_3_ (69–72 %) and 3 mL HClO_4_ (70 %) on a micro Kjeldahl digestion apparatus at 350 °C for 200 min. After cooling, the digest was filtered and diluted to 25 mL with deionized water. Triplicate digestions were carried out for each sample. The blank solutions were prepared by digesting the mixture of reagents following the same digestion procedure and diluted to 25 mL with deionized water.

### Determination of metals in the tobacco samples

Four points calibration curves were established by running the standard solutions (10 mg/L) in flame AAS. Immediately after calibration, the sample solutions were aspirated into the AAS instrument and direct readings of the metal concentrations was recorded. Three replicate determinations were carried out on each sample. The same analytical procedure was employed in the determination of elements in each six digested blank.

### Procedure of spiking

To confirm the efficiency of developed optimized procedures, spiking experiments in which known volume and concentration of standard solutions, were employed. From the stock solution (1000 mg/L) an intermediate standard solutions (10 mg/L) were prepared for all the metals. 13 μL of Cd, 30 μL of Cr, Ni, Pb, 50 μL of Cu, and 70 μL of Zn from 10 mg/L solutions were added to 0.50 g tobacco leaves collected from Shewa Robit. The same amounts of solutions were added to processed tobacco sample collected from Billate and Nyala except the volume of Cd added was increased to 30 μL (since the amount Cd was increased in samples). Then samples were digested and analyzed with the previously optimized procedures.

## Results and discussion

### Optimization of digestion procedure

Concentrated perchloric acid is a powerful oxidizing agent when hot. However, due to the risk of explosion, perchloric acid was used in mixture with nitric acid which serves not only to dilute the perchloric acid but also to ensure the easily oxidizable compounds are broken by reaction with nitric acid first at low temperature before the perchloric acid starts to exert its oxidizing power at 160 °C (Bock 1979). It is recommended that the sample size should be less than 1 g for the reason of safety when perchloric acid used for digestion (Bock 1979). Refluxing is compulsory, when a sample is decomposed by open ashing to determine volatile trace elements like Cd (Bock 1979). Using these reagents and 0.5 g sample different digestion methods were tested and the procedure that produce clear solution, consumed minimal reagent volumes and shorter digestion time, with acceptable sample masses of tobacco samples was selected from the tested alternatives (Bock 1979; Health Canada 1999). Optimization of the digestion procedure involved some changes of parameters such as reagent volume, digestion temperature and digestion time. Based upon above listed criteria, the optimal digestion procedure chosen was the one that fulfilled the stated criteria for complete digestion of 0.5 g of the dry sample powders, with 3 mL HNO_3_ (69–72 %) and 3 mL HClO_4_ (70 %) for total of 3:30 h. The mixture was heated smoothly for 10 min by adjusting the temperature to 150 °C. After 10 min when the evolution of oxides of nitrogen ceased the mixture was heated strongly by adjusting the temperature 350 °C. The procedures that required higher reagent volume, longer digestion time, and which resulted in the formation of turbid digests and colored digest solutions were rejected.

### Precision of results

Precision can be determined by standard deviation, variance, coefficient of variance, relative standard deviation, and range of series measurements (Miller and Miller 2000). In this study the precision of the results were evaluated by the pooled standard deviation and relative standard deviation of the results of triplicate samples and three reading (n = 9) obtained for each sample. The result of analysis was reported with corresponding standard deviation at 95 % confidence limit and relative standard deviation. It can be seen that the values of percentage relative standard deviations (% RSD) are less than 10 % for all the mean concentrations. This shows the precision of the results obtained by this method is good and acceptable.

### Validation of optimized procedure

The efficiency of the optimized procedure was evaluated by analyzing the digests of spiked samples for both raw tobacco leaves and processed tobacco samples (Miller and Miller 2000). The recoveries of metals in the spiked tobacco samples were in the range 88.3–107 %. These values are within the acceptable range for analyses of biological samples such as plants. The results are given in Table 1. Generally, good recoveries were obtained for all the metals. As can be seen from Table 1, percentage recoveries of all metals in all samples are within the range of 100 ± 10 %. Thus, on the average good recoveries were obtained for all elements in all the samples validating that the optimized procedure has good accuracy.

**Table 1:** Analytical results obtained for validation of the optimized procedure after spiking with standard solutions

Metal	Type of tobacco sample	Amount in unspiked sample (μg/g)	Amount added (μg/g)	Amount in spiked sample (μg/g)	(%) Recovery
Cd	Shewa Robit raw tobacco leaves	1.30 ± 0.04	0.26	1.54 ± 0.07	92.3 ± 6.0
	Billate processed tobacco leaves	1.45 ± 0.023	0.6	2.03 ± 0.03	96.7 ± 1.9
	Nyala processed tobacco leaves	1.55 ± 0.23	0.6	2.11 ± 0.06	93.3 ± 3.6
Cr	Shewa Robit raw tobacco leaves	1.45 ± 0.11	0.6	1.99 ± 0.12	90.0 ± 7.8
	Billate processed tobacco leaves	1.65 ± 0.08	0.6	2.23 ± 0.03	96.7 ± 1.7
	Nyala processed tobacco leaves	1.62 ± 0.11	0.6	2.19 ± 0.08	95.0 ± 4.5
Cu	Shewa Robit raw tobacco leaves	7.30 ± 0.19	1.0	8.34 ± 0.02	104 ± 0.3
	Billate processed tobacco leaves	9.80 ± 0.04	1.0	10.8 ± 0.08	102 ± 1
	Nyala processed tobacco leaves	8.95 ± 0.31	1.0	10.0 ± 0.24	107 ± 3.1
Ni	Shewa Robit raw tobacco leaves	1.96 ± 0.08	0.6	2.49 ± 0.02	88.3 ± 0.9
	Billate processed tobacco leaves	2.35 ± 0.19	0.6	2.91 ± 0.08	93.8 ± 3.6
	Nyala processed tobacco leaves	4.70 ± 0.04	0.6	5.25 ± 0.25	91.7 ± 6.1
Zn	Shewa Robit raw tobacco leaves	33.2 ± 1.9	1.4	34.6 ± 0.25	104 ± 0.9
	Billate processed tobacco leaves	101 ± 0.4	1.4	103 ± 0.4	106 ± 0.5
	Nyala processed tobacco leaves	79.3 ± 0.77	1.4	80.6 ± 0.37	94.3 ± 0.6

### Concentration of metals in the raw tobacco leaves

Tobacco plant is known to easily absorb heavy metals from soil and accumulate them in the leaves. In this study (Table 2) highest concentration of Zn was determined in the raw tobacco leaves samples from both sample sites, Billate and Shewa Robit. However, higher average content of Zn (53.7 μg/g dry mass) was determined in tobacco leaves from Billate than Shewa Robit (33.2 μg/g dry mass). Except the amount Zn, all the metals were higher in tobacco leaves from Shewa Robit than Billate. Pb was found to be lower than the detection limit of the instrument in both the tobacco leaves samples. Ni was determined lower than instrument detection limit in the tobacco leaves from Billate.

**Table 2:** Metal concentration (mean ± SD) in Ethiopian raw and processed tobacco leaves

Type of tobacco sample	Concentration of metals (μg/g)					
	Cd	Cu	Cr	Ni	Pb	Zn
Billate raw tobacco leaves	1.20 ± 0.05	4.38 ± 0.11	ND	ND	ND	53.7 ± 0.96
Shewa Robit raw tobacco leaves	1.30 ± 0.04	7.30 ± 0.19	1.45 ± 0.11	1.96 ± 0.08	ND	33.2 ± 1.9
Billate processed tobacco leaves	1.45 ± 0.02	9.80 ± 0.04	1.65 ± 0.08	2.35 ± 0.19	ND	101 ± 0.4
Shewa Robit processed tobacco leaves	1.90 ± 0.05	12.8 ± 0.11	1.75 ± 0.08	2.20 ± 0.05	ND	83.8 ± 0.4
Nyala processed tobacco leaves	1.55 ± 0.07	8.95 ± 0.31	1.62 ± 0.11	4.70 ± 0.04	ND	79.3 ± 0.77

*ND* not detected

The results of this study showed that the metal contents of tobacco leaves varied with the geographical origin in which the tobacco plant grows. Even though there are no industrial activities in both areas of sampling, the natural weathering of rocks, agricultural activities like using fertilizer, herbicide, and water for irrigation could contribute to these concentrations of metals determined in tobacco leaves. A comparison of the metal contents in the tobacco leaves in this study showed that Zn contents are in higher amounts in the samples compared to the other heavy metals. A relatively higher amount of Zn in the leaves from both areas could be due to its higher natural abundance in the soil. Both areas of sampling were using phosphate fertilizer, which has significant contribution to metals concentration in tobacco leaves. Concentrations of other heavy metal in phosphate fertilizers vary considerably, depending on the phosphate rock source (Peuarossa et al. 1990).

In comparison to Billatte, Shewa Robit is more exposed to human activity and the farms were used for long time, which could have significant contribution to gradual accumulation of metals in tobacco farms through agricultural activities. This could be suggested as one of the reasons for the higher concentration of most metals determined in Shewa Robit than Billate. As indicated previously metals like Ni, Pb, and Cr in Billate leaves sample were below the detection limit of the instruments.

### Concentration of heavy metals in the processed tobacco leaves

The average metal concentrations in processed tobacco leaves whose origin was from Billate were Cd (1.45), Cu (9.8), Ni (2.35), Cr (1.65), and Zn (101) μg/g dry mass. The amounts of metals determined in the processed tobacco leaves whose origin was from Shewa Robit were Cd (1.9), Cu (12.8), Ni (2.2), Cr (1.75), Zn (83.8) μg/g dry mass. In the same way Cd (1.55), Cu (8.95), Cr (1.62), Ni (4.7), Zn (79.3) μg/g dry mass concentrations were found in Nyala (Table 2). Here large amount Zn was observed in processed tobacco sample collected from Billate in comparison to the others. The concentration of Ni was found to be higher in processed tobacco sample from Billate than the processed tobacco sample from Shewa Robit. In all the processed tobacco samples, the amount of Pb was found to be lower than the detection limit of the instruments.

Nyala type processed tobacco sample is different from other two processed tobacco in that it is the mixture of tobacco leaves from Shewa Robit and Billate, which account about 45–50 %, and imported leaves from different country (Brazil, Zimbabwe, and India). In comparison to both processed samples from Billate and Shewa Robit higher Ni (4.7 μg/g dry mass) concentration was determined in Nyala sample. This concentration of Ni in Nyala could be suggested to come from tobacco leaves, which were imported.

### Comparison of metals in the raw tobacco leaves and processed tobacco samples

The variation in composition of metals in the raw and processed tobacco leaves was observed for all the detected metals (Table 2).

This change in concentration of metals could be due to treatments and handling of tobacco leaves starting from harvesting to the cigarette manufacturing in the factory. During harvesting, transportation from the farm to the site of curing, transportation from site of curing to the factory and system of the storage could make leaves of tobacco be contaminated with dusts and soil, which contain these metals. The processes of packing and packing materials, curing system, treatments in the factory and chemical additives (casing activities) in the manufacturing, could have contribution for the contamination the tobacco leaves with the metals.

The other factor that made the large difference in concentration of metals could be: during the collection of tobacco leaves for analysis, in this study, the collected samples were washed with tape water and rinsed with distilled-deionized water. Since there is no such treatment in processed tobacco or in manufacturing of cigarette, the extraneous substances including soil and dust particles, and foliar spray residues could introduce extra metal contamination. Particularly tobacco leaves from the lower part were highly contaminated with soil. Therefore, metals from the soil that were deposited on the leaves could contribute to the high level of metals in the processed tobacco.

### Comparison of metals concentration in Ethiopian tobacco with literature values

Many researchers have reported the concentration of metals in cigarette tobacco as well as tobacco leaves. Moulin et al. (2006) analyzed 755 tobacco’s leaves samples during 2001–2003 and found that cadmium concentrations in the samples ranged from 0 to 6.78 μg/g dry mass. The report also indicated that Cd contents of flue cured tobacco leaves as India (0.33 ± 0.13), France (1.46 ± 1.35) and processed one from USA (0.51 ± 0.05) μg/g dry mass.

There are also other literatures which reported the contents of some metals such as Cu (14.9–21.1), Zn (51–84), Ni (<1 μg/g dry mass), in flue cured tobacco leaves 24–33 μg/g dry mass of Zn (Tso 1973); the concentration of nickel in cigarettes (2.32–4.20 μg/g dry mass) and in tobacco leaves (2.20–4.91 μg/g dry mass) (Stojanovic et al. 2004); the average concentration of Cd in both tobacco leaves and cigarettes in Mexican produced-tobacco (4.41 ± 0.67 and 2.65 ± 0.99 μg/g dry mass respectively) (Saldivar et al. 1991).

As compared the report of Murty et al. (1986) (Table 3) the concentration of Cd in present study flue cured tobacco was higher than flue cured tobacco of India, New Zealand and within the range of other countries (America, Germany, and Canada) flue cured tobacco concentration. Generally the level of Cd in present study was within the range of the literature values, which can range from 0 to 6.78 (Moulin et al. 2006). However, in comparison with the flue-cured leaves from India and France, Cd content in present study was found to be higher than that of Indians’ and lower than Frances’ flue cured leaves (Moulin et al. 2006). The concentration of Ni in the present study was found to be within the range of literature value between 1 to 4.91 μg/g dry mass (Stojanovic et al. 2004; Tso 1973). The concentration of Cu is lower than the literature value which ranges from 14.9–21.14 μg/g. The level of Zn in the tobacco leaves in this study is also within the range of literature value, which ranges from 24 to 81 μg/g dry mass. Precise reported information was not obtained on the content of Cr in tobacco leaves from literatures.

**Table 3:** Comparison of levels of Cd and Pb (μg/g dry mass) in Ethiopian raw tobacco leaves (present study) with literature values (Murty et al. 1986)

Metals	Ethiopia (present study)	India	America	Germany	New Zealand	Canada
Cd	1.20–1.30	0.218–0.494	1.7–2.9	1.07–2.3	0.23–0.56	1.25–7.02
Lead	ND	0.311–0.416	0–200	2.4–4.3	0.48–0.55	0.8–9.15

*ND* not detected

There are also different reports of metal contents in processed tobacco from different countries, some of these are Vastarella et al. (2003), Ei-Amri et al. (1989), Zhang et al. (2005) and Nnorom et al. (2005) (Table 4).

**Table 4:** Comparison of metal concentration (μg/g dry mass) in the Ethiopian processed tobacco leaves (present study) with literature data (Zhang et al. 2005; Moulin et al. 2006; Ei-Amri et al. 1989)

Metals	Ethiopia (mean, present study)	Ethiopia (Nyala, present study)	UK	France	Belgium	Italy	Germany	Japan	References
Cd	1.67	1.55	1.62	1.59	1.22	1.96	1.96	1.04	Zhang et al. (2005)
Ni	2.27	4.70	5.62	8.91	9.44	8.60	9.11	3.24	Zhang et al. (2005)
Pb	ND	ND	14.3	15.6	14.7	22.0	17.2	NR	Zhang et al. (2005)
Cr	1.70	1.62	0.11	0.05	0.06	0.07	0.07	NR	Zhang et al. (2005)
Zn	92.7	79.3	NR	NR	NR	NR	NR	75.8	Zhang et al. (2005)

Metal	Ethiopia (present study)	Cameroun	France	Germany	Switzerland	UK	USA		Reference
Cd	1.45–1.90	1.30	2.30	1.80	1.27	1.34	1.60		Moulin et al. (2006)

Metals	Ethiopia (present study)	Libya	India	USA	Iran	Yugoslavia			References
Cr	1.62–1.75	4.55–4.85	3.0–8.2	0.24–6.3	4.3–6.2	NR			Ei-Amri et al. (1989)
Cu	8.95–12.8	32.8–41.1	NR	NR	NR	18.9			Ei-Amri et al. (1989)
Zn	79.3–101	63.5–95.8	15–31	4.1–54	51–56	51			Ei-Amri et al. (1989)

*ND* not detected, *NR* not reported

As presented in Table 4 the Cd concentration of present study is within the range of minimum concentration determined in Japan’s cigarette (Zhang et al. 2005) and maximum concentration obtained in the France cigarette (Zhang et al. 2005) (Table 4). The concentration of Pb obtained by this method is less than all the literature report. Similarly Ni in processed Shewa Robit and Billate is less than all other literature report. However, the Ni concentration determined in Nyala is within the range minimum report (2.32 μg/g) (Stojanovic et al. 2004) and maximum content of Germany’s (9.11 μg/g dry mass) cigarette (Saldivar et al. 1991).

Comparative results given Table 4 revealed that the Zn concentration in present study is found to be higher than the literature value. In contrast to Zn, the amount of Cu is found to be lower than the literature values. The concentration of lead in present study is lower than its concentration in processed tobacco from other countries (Table 4). It has been demonstrated that most of the lead in green plant parts originate from deposition of air borne lead from automotive sources (Murty et al. 1986) and thus the lead content of tobacco leaves in this study can be expected to be low as such occurrences are minimum in Ethiopia. Even the lead in the soil is not in soluble form to be available to plant as compared to other metals.

In general, the concentrations of metals observed were more or less comparable with the reported literature values. However, relatively lower concentrations of Cu were observed in this study in comparison to the reported values.

## Conclusions

An efficient digestion procedure was developed and validated through recovery studies. The optimal digestion procedure allowed the use of acids with minimum volumes leading to reduced blank concentration, and lowers the method detection limit. Furthermore, this condition allowed most elements determined with greater precision and accuracy.

This investigation revealed the dependence of metal accumulation in tobacco leaves on the geographical origin in which tobacco plant grows. The investigation has indicated the presence of heavy metals (Cu, Zn, Cd, Ni, Cr), provided baseline data for comparison, give good awareness for general people (smokers and non-smokers), Ethiopian Tobacco Enterprise and Health Organizations. In addition, this study revealed comparability of the metal component of Ethiopian tobacco with other countries’ tobacco. The concentrations of most of the metals detected are found to be within the range of literature values, except copper, which was found to be slightly lower than literature values. Pb was not detected in the Ethiopian tobacco leaves which make the Ethiopian tobacco free this toxic heavy metal.

Even though there is no industrial activity and no contaminated sewage sludge application in both the Ethiopian sample sites (Billate and Shewa Robit), the Cd concentration in Ethiopian tobacco was found to be comparable with industrialized countries and greater than that naturally available in the soil. Fertilizer could be the main contributor for such un-expected concentration of Cd in these un-industrialized and free of human impact sample sites.

This study revealed that there was large difference between the heavy metal content of raw tobacco leaves and processed tobacco. The large increment in composition of heavy metals in processed tobacco was observed for all the detected metals. This indicated that the metal contents that determined in cigarette are not only the content of raw leaves itself but also the metal originated from contamination of raw leaves during the process, starting from harvesting to cigarette manufacturing. Therefore, to control further contamination of tobacco leaves with toxic metals, well treatments in handling, transportation and storage of tobacco leaves are recommended.

## References

[CR1] Adeyeye EI (2005). Trace metals in soil and plants from Fadama Farm in Ekitistat, Nigeria. Bull Chem Soc Ethiop.

[CR2] Ajab H, Yaqub A, Malik SA, Junaid M, Yasmeen S, Abdullah MA (2014). Characterization of toxic metals in tobacco, tobacco smoke, and cigarette ash from selected imported and local brands in Pakistan. Sci World J.

[CR3] Angelova V, Ivanov K, Ivanova R (2004). Effect of chemical forms of lead, cadmium, and zinc in polluted soils on their uptake by tobacco. J Plant Nutr.

[CR4] Atlabachew M, Chandravanshi BS, Redi M (2010). Concentration levels of essential and non-essential metals in Ethiopian khat (*Catha edulis* Forsk). Biol Trace Elem Res.

[CR5] Atlabachew M, Chandravanshi BS, Redi M (2011). Profile of major, minor and toxic metals in soil and khat (*Catha edulis* Forsk) cultivars in Ethiopia. Trends Appl Sci Res.

[CR6] Baldwin DR, Marshall WJ (1999). Heavy metal poisoning and its laboratory investigation. Ann Clin Biochem.

[CR7] Bock R (1979). Decomposition method in analytical chemistry.

[CR8] Campbell RCJ, Stephens WE, Meharg AA (2014) Consistency of arsenic speciation in global tobacco products with implications for health and regulation. Tob Induc Dis 12: 24, http://www.tobaccoinduceddiseases.com/content/12/1/24 10.1186/s12971-014-0024-5 PMC4275931 25540607

[CR9] Chang MJ, McDaniel RL, Naworal JD, Self DA (2002). A rapid method for the determination of mercury in mainstream cigarette smoke by two-stage amalgamation cold vapor atomic absorption spectrometry. J Anal At Spectrom.

[CR10] Ei-Amri FA, Saleh AI, Ei-Gnidy BA (1989). Determination of trace element concentrations of Libyan chewing and cigarette tobacco by instrumental neutron activation analysis. J Radioanal Chem.

[CR11] Eneji IS, Salawu OW, Sha’Ato R (2013). Analysis of heavy metals in selected cigarettes and tobacco leaves in Benue State, Nigeria. World J Med Med Sci Res.

[CR12] FAO (Food and Agricultural Organization) (1995). Country information brief, agricultural production and diversification programmes, review of food and cash crops production.

[CR13] FAO (Food and Agriculture Organization of the United Nations) (2003). Projections of tobacco production, consumption and trade to the year 2010.

[CR14] Gebre Selassie S, Gebre A (1996) Rapid assessment of drug abuse in Ethiopia, UNODC—Bulletin on Narcotics—1996, Issue 1-004, pp 53–63 9839035

[CR15] Geto A, Amare M, Tessema M, Admassie S (2012). Voltammetric determination of nicotine at poly(4-amino-3-hydroxynaphthalene sulfonic acid)-modified glassy carbon electrode. Electroanalysis.

[CR16] Golia EE, Dimirkou A, Mitsios IK (2008). Levels of heavy metals pollution in different types of soil of central Greece. Bull Environ Contam Toxicol.

[CR17] Grant CA, Buckley WT, Bailey LD, Selles F (1998). Cadmium accumulation in crops. Can J Plant Sci.

[CR18] Health Canada (1999) Determination of Ni, Pb, Cd, Cr, As, Se and Hg in whole tobacco, tobacco control programme. Official Method, T-306, No. 1, pp 1–16

[CR19] Iyengar GV, Subramanian KS, Woittiez JRW (1998). Elemental analysis of biological samples.

[CR20] Jarup L, Berglund M, Elinder CG, Nordberg G, Vahter M (1998). Health effects of cadmium exposure—a review of the literature and a risk estimate. Scand J Work Environ Health.

[CR21] Jones JB, Wolf B, Mills H (1991). Plant analysis handbook.

[CR22] Kaličanin B, Velimirović D (2012). Potentiometric stripping analysis of zinc, cadmium and lead in tobacco leaves (*Nicotiana Tabacum* L.) and soil samples. Int J Electrochem Sci.

[CR23] Karaivazoglou NA, Tsotsolis NC, Tsadilas CD (2007). Influence of liming and form of nitrogen fertilizer on nutrient uptake, growth, yield, and quality of Virginia (flue-cured) tobacco. Field Crops Sci.

[CR24] Kassa H, Geto A, Admassie S (2013). Voltammetric determination of nicotine in cigarette tobacco at electrochemically activated glassy carbon electrode. Bull Chem Soc Ethiop.

[CR25] Kowalski R, Wierciński J (2009). Mercury content in smoke and tobacco from selected cigarette brands. Ecol Chem Eng S.

[CR26] Lazarević K, Nikolić D, Stošić L, Milutinović S, Videnović J, Bogdanović D (2012). Determination of lead and arsenic in tobacco and cigarettes: an important issue of public health. Cent Eur J Public Health.

[CR27] Lecours N, Almeida GEG, Abdallah JM, Novotny TE (2012). Environmental health impacts of tobacco farming: a review of the literature. Tobacco Control.

[CR28] Lugon-Moulin N, Ryan L, Donini P, Rossi L (2006). Cadmium content of phosphate fertilizers used for tobacco production. Agron Sustain Dev.

[CR29] Lugon-Moulin N, Martin F, Krauss MR, Ramey PB, Rossi L (2008). Arsenic concentration in tobacco leaves: a study on three commercially important tobacco (*Nicotiana tabacum* L.) types. Water Air Soil Pollut.

[CR30] Maier QEA, Griepink B (1995). Quality assurance for environmental analysis.

[CR31] Maruyama Y, Komiya K (1973). Determination of copper, arsenic and mercury in tobacco leaves by neutron activation analysis. Radioisotopes.

[CR32] Miller JN, Miller JC (2000). Statistics and chemometrics for analytical chemistry.

[CR33] Moulin N, Martin F, Krauss F, Ramey PB, Rossi L (2006). Cadmium concentration in tobacco from different countries and its relationship with other elements. Chemosphere.

[CR34] Murty KSN, Tjell JC, Gopalachari NC (1986). Lead and cadmium content of Indian flue-cured tobacco. Plant Soil.

[CR35] National Tobacco Enterprise (NTE) (2006). Draft of strategic plan of National Tobacco Enterprise (NTE).

[CR36] Nnorom IC, Osibanjo O, Oji-nnorom CG (2005). Cadmium determination in cigarettes available in Nigeria. Afr J Biotechnol.

[CR37] Nordberg G, Nogawa K, Nordberg M, Friberg L (2007). Handbook on toxicology of metals.

[CR38] Peuarossa BF, Malorgio L, Lubrano F, Petnine G (1990). Phosphatic fertilizers as source of heavy metals in protected cultivation. Commun Soil Sci Plant Anal.

[CR39] Piadé J-J, Jaccard G, Dolka C, Belushkin M, Wajrock S (2015). Differences in cadmium transfer from tobacco to cigarette smoke, compared to arsenic or lead. Toxicol Rep.

[CR40] Rothwell K, Yorkshire K (1999). Health effects of interactions between tobacco uses and exposure to other agents.

[CR41] Saldivar L, Luna M, Reyes E, Soto R (1991). Cadmium determination in Mexican-produced tobacco. Environ Res.

[CR42] Sharma P, Dubey RS (2005). Lead toxicity in plants. Braz J Plant Physiol.

[CR43] Smith CJ, Doolittle DJ (1999). An international literature survey of “IARC Group I Carcinogens” reported in mainstream cigarette smoke. Food Chem Toxicol.

[CR44] Stojanovic D, Nikic D, Lazarevic K (2004). The level of nickel in smoker’s blood and urine. Cent Eur J Public Health.

[CR45] Suzuki T, Shishido S, Urushiyama K (1976). Mercury in cigarettes. Tohoku J Exp Med.

[CR46] Taebunpakul S, Liu C, Wright C, McAdam K, Heroult J, Braybrook J, Goenaga-Infante H (2011). Determination of total arsenic and arsenic speciation in tobacco products: from tobacco leaf and cigarette smoke. J Anal At Spectrom.

[CR47] Tassew Z, Chandravanshi BS (2015). Levels of nicotine in Ethiopian tobacco leaves. SpringerPlus.

[CR48] Tso TC (1973). Physiology and biochemistry of tobacco plant.

[CR49] Vastarella W, Mola M, Caputi M (2003) Determination of trace metals in main and sidestream smoke by ICP-AEP: a procedure for recovery and analysis. Annual report about the Research Project, Napoli, Italian Tobacco Agency

[CR50] Verma S, Yadav S, Singh I (2010). Trace metal concentration in different Indian tobacco products and related health implications. Food Chem Toxicol.

[CR51] Zerihun A, Chandravanshi BS, Debebe A, Mehari B (2015). Levels of selected metals in leaves of *Cannabis sativa* L. cultivated in Ethiopia. SpringerPlus.

[CR52] Zhang C, Miura J, Nagoas Y (2005). Determination of cadmium, zinc, nickel and cobalt in tobacco by reversed-phase high-performance liquid chromatography with 2-(8-quinolylazo)-4,5-di phenylimidazole as chelating reagent. Anal Sci.

[CR53] Zhangyu C, Yang G-Y, Shuhua W, Ling L, Qingde S (2004). Simultaneous determination of tin, nickel, lead and mercury in tobacco and tobacco additives by microwave digestion and RP-HPLC followed by on-line column enrichment. J Chin Chem Soc.

